# Resection of a Large Intra-Abdominal Desmoid Tumor with Preoperative Superior Mesenteric Artery Balloon Catheter Placement: A Case Report

**DOI:** 10.70352/scrj.cr.26-0550

**Published:** 2026-10-07

**Authors:** Kentaro Suzuki, Yuki Sekido, Chikako Kusunoki, Tsuyoshi Hata, Ryota Mori, Mitsunobu Takeda, Masaaki Miyo, Atsushi Hamabe, Takayuki Ogino, Norikatsu Miyoshi, Mamoru Uemura, Hidetoshi Eguchi

**Affiliations:** 1 Postgraduate Clinical Training Center, The University of Osaka Medical Hospital, Suita, Osaka, Japan; 2 Department of Gastroenterological Surgery, The University of Osaka Graduate School of Medicine, Suita, Osaka, Japan

**Keywords:** desmoid tumor, desmoid-type fibromatosis, intra-abdominal, superior mesenteric artery, balloon occlusion, interventional radiology, large desmoid tumor, mesentery

## Abstract

**INTRODUCTION:**

Large tumors occupying the abdominal cavity can hinder surgical exposure, particularly of the posterior aspect, increasing the risk of arterial bleeding. Because arterial hemostasis is difficult, hemodynamic instability may occur. This report describes the safe use of preoperative balloon catheter placement in the superior mesenteric artery (SMA), the tumor’s primary feeding vessel, to control arterial bleeding through on-demand intraoperative occlusion.

**CASE PRESENTATION:**

A 40-year-old woman with a history of 2 cesarean sections presented with abdominal distension. An intra-abdominal mass was identified at another institution, and she was referred to our hospital. Contrast-enhanced abdominal CT revealed a large intra-abdominal mass supplied primarily by jejunal branches of the SMA. US-guided biopsy confirmed desmoid tumor. Chemotherapy was also considered; however, debulking surgery followed by chemotherapy was planned to relieve abdominal symptoms. Because the large tumor was expected to limit surgical exposure and increase the risk of intraoperative bleeding, an SMA balloon catheter was electively placed the day before surgery. During surgery, the balloon was inflated on demand by the anesthesiologist at the surgeon’s direction before maneuvers carrying a risk of arterial bleeding. Tumor involvement of the small bowel mesentery, transverse mesocolon, small intestine, and duodenum required *en bloc* resection. As SMA involvement was confined to the segment distal to the ileocolic artery bifurcation, complete macroscopic resection was feasible while preserving approximately 200 cm of the small bowel. The resected specimen weighed 5660 g. Immunohistochemistry showed nuclear β-catenin positivity, confirming the diagnosis of desmoid tumor. Negative surgical margins achieved R0 resection. One year later, MRI detected a 1.5-cm intra-abdominal recurrence in the right upper abdomen. Chemotherapy achieved stable disease after 7 months but was discontinued because of nausea and vomiting. At 3 years postoperatively, no disease progression has been observed.

**CONCLUSIONS:**

In large intra-abdominal desmoid tumors with anticipated limited surgical exposure, preoperative SMA balloon catheter placement may facilitate arterial-bleeding control and improve surgical safety.

## Abbreviations

CTCAE
: Common Terminology Criteria for Adverse Events
FAP
: familial adenomatous polyposis
ICA
: ileocolic artery
IVR
: interventional radiology
MTX
: methotrexate
SMA
: superior mesenteric artery
VBL
: vinblastine

## INTRODUCTION

A desmoid tumor is a fibroblastic neoplasm arising from the fascia or aponeurosis. Although histologically benign, it is locally aggressive and has a high local recurrence rate.^1)^ Large tumors often limit surgical exposure, particularly in the posterior aspect, increasing the risk of arterial bleeding.

FAP, mechanical stimulation such as surgery or trauma, and pregnancy are established risk factors.^2)^ Although active surveillance has become the first-line strategy for asymptomatic cases, surgical resection remains indicated for patients with symptoms, functional impairment, pain, perforation, obstruction, or progressive tumor growth.^3)^

Intra-abdominal desmoid tumors are particularly difficult to resect because they frequently involve the mesentery or major vessels, increasing the risk of massive intraoperative arterial bleeding.^4)^

This report describes successful resection of a large intra-abdominal desmoid tumor supplied by the jejunal branches of the SMA. Elective preoperative SMA balloon catheter placement via IVR enabled on-demand intraoperative arterial occlusion and facilitated safe tumor resection. The relevant literature is also reviewed.

## CASE PRESENTATION

The patient was a 40-year-old woman who had experienced abdominal distension for 2 years. Initial evaluation at another institution revealed no abnormalities. She was later admitted for pyelonephritis, during which abdominal CT incidentally identified an intra-abdominal mass, prompting referral to our hospital for further evaluation and treatment.

Her medical history included 2 cesarean sections, the most recent approximately 2 years before her first presentation to the Department of Gastroenterological Surgery. Her family history was notable for lung cancer in her grandfather. She had no history of FAP or Gardner syndrome and did not smoke or consume alcohol.

On physical examination, her height was 155 cm and her weight was 66 kg (BMI 27.5). The abdomen was markedly distended with a palpable mass but no tenderness. Laboratory tests showed a white blood cell count of 3780/μL, hemoglobin of 14.1 g/dL, platelet count of 27.5 × 10^4^/μL, carcinoembryonic antigen of 1 ng/mL, and carbohydrate antigen 19-9 of 12.8 U/mL. Tumor markers were not elevated.

Contrast-enhanced abdominal CT demonstrated a 25 × 23 × 17-cm intra-abdominal mass supplied primarily by the first and second jejunal branches of the SMA (**Fig. 1A** and **1C**). No lymph node or distant metastases were identified.

**Fig. 1:**
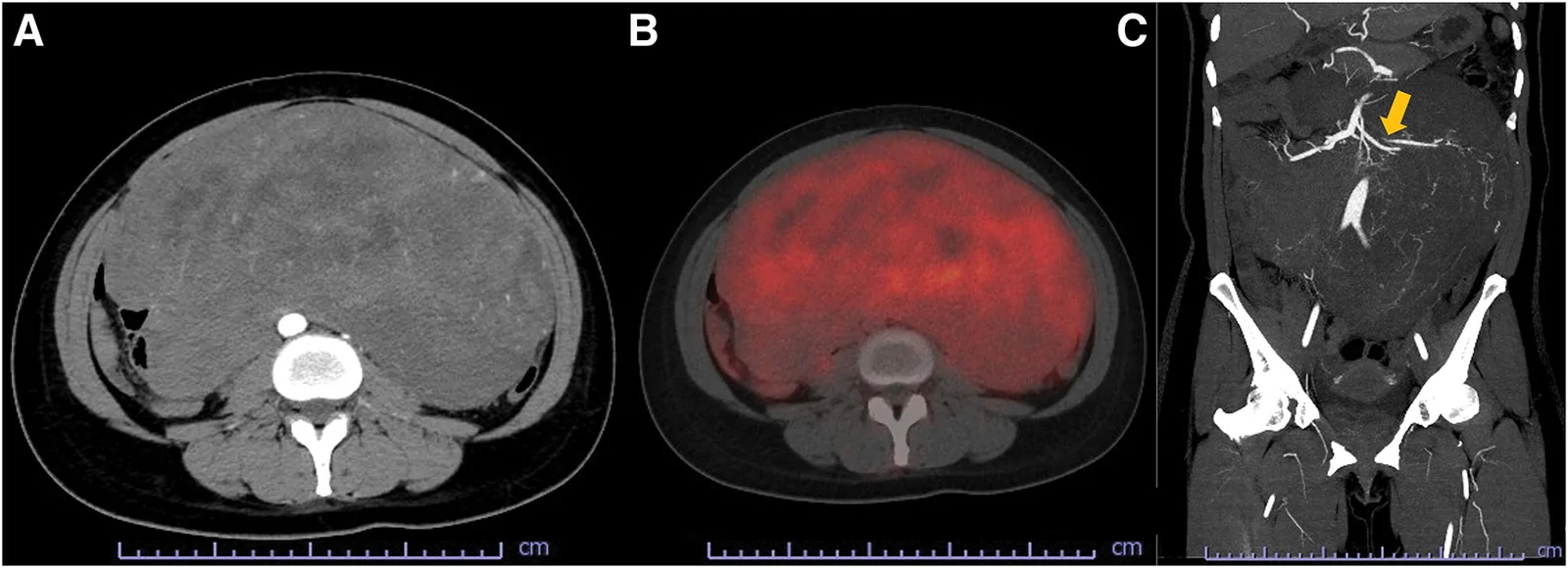
Preoperative radiological findings. (**A**) Contrast-enhanced abdominal CT reveals a large mass occupying the abdominal cavity. (**B**) PET-CT demonstrates diffuse fluorodeoxyglucose uptake throughout the tumor. (**C**) Preoperative abdominal angiography. The arrow indicates a jejunal artery, a branch of the superior mesenteric artery, representing the primary feeding vessel of the tumor.

PET-CT showed diffuse fluorodeoxyglucose uptake with a maximum standardized uptake value of 4.61 (**Fig. 1B**). MRI demonstrated low signal intensity on T1-weighted images and heterogeneous high signal intensity with cord-like low-signal areas suggestive of vascular structures on T2-weighted images (**Fig. 2A** and **2B**).

**Fig. 2:**
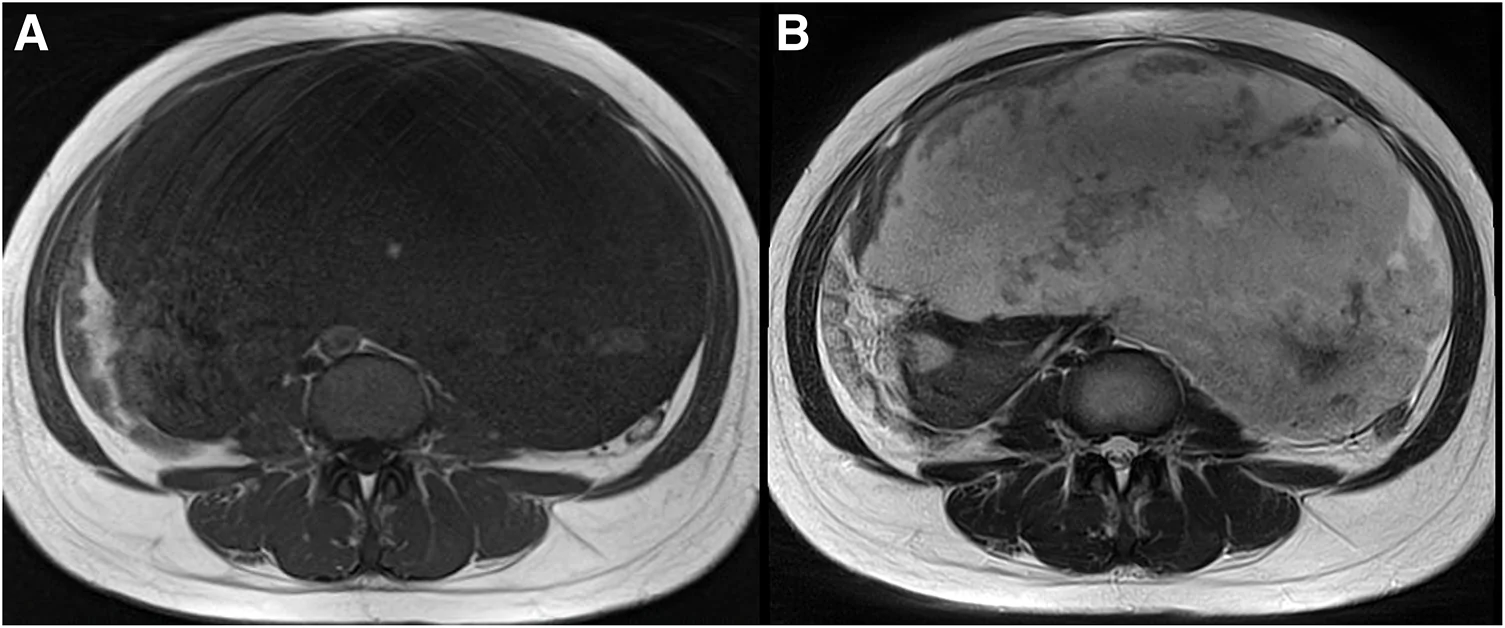
Preoperative MRI findings. (**A**) T1-weighted image. (**B**) T2-weighted image.

US-guided biopsy revealed intersecting fascicles of spindle-shaped cells without mitotic figures or necrosis. Immunohistochemistry demonstrated nuclear positivity for β-catenin and negativity for c-kit, DOG1, CD34, α-SMA, desmin, and S-100, excluding gastrointestinal stromal tumor and confirming the diagnosis of desmoid tumor.

Systemic chemotherapy with MTX plus VBL, followed by elective resection once a response had been assessed, was also considered; however, her abdominal symptoms were severe.

On preoperative contrast-enhanced CT, the level of SMA involvement relative to the ICA bifurcation could not be determined because of the mass effect of the 25-cm tumor. As the tumor encased the SMA, whether complete resection with negative margins was anatomically feasible depended on the level of SMA involvement, which could not be established preoperatively. Accordingly, initial debulking surgery, followed by chemotherapy for residual disease, was planned. Given the tumor size and anticipated risk of intraoperative arterial bleeding, a balloon catheter (Selecon MP Catheter II, 5Fr; Terumo, Tokyo, Japan) was electively placed at the SMA origin via IVR the day before surgery.

Angiography confirmed complete SMA occlusion with balloon inflation to 0.15 mL, demonstrating the feasibility of intraoperative arterial occlusion (**Fig. 3**). A preoperative multidisciplinary briefing involving the gastrointestinal surgery and anesthesiology teams assigned balloon control to the anesthesiologist under the lead surgeon’s direction.

**Fig. 3:**
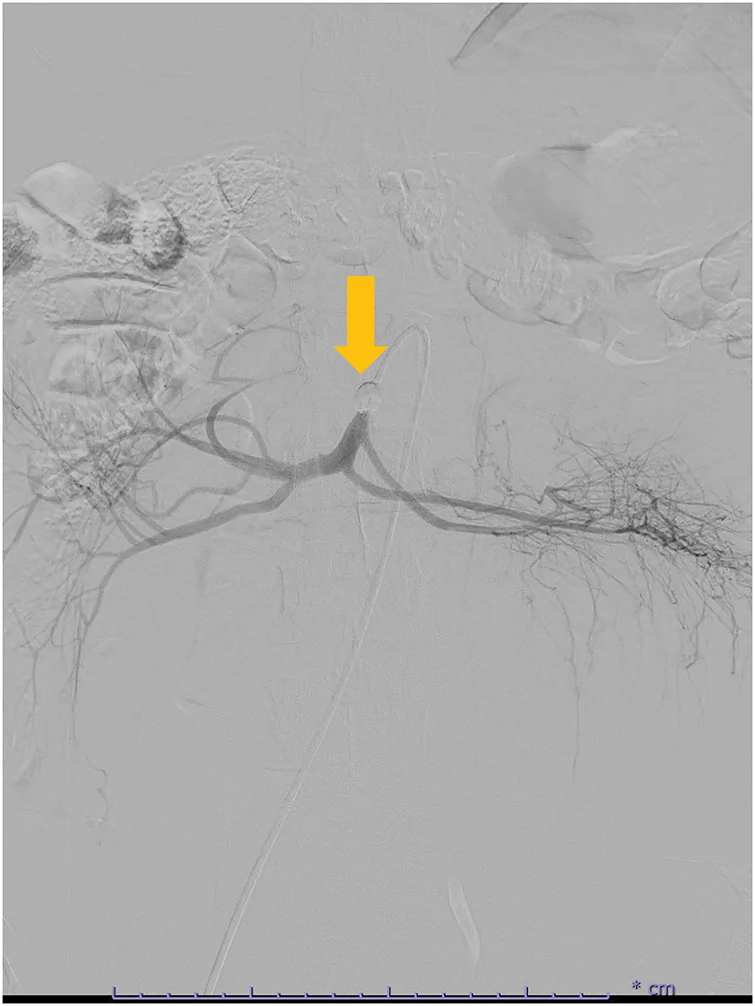
Preoperative angiography confirming SMA balloon catheter occlusion. The arrow indicates the balloon catheter positioned at the root of the SMA. A balloon catheter (Selecon MP Catheter II, 5 Fr; Terumo, Tokyo, Japan) was electively placed via the right femoral approach by interventional radiology the day before surgery. Inflation with 0.15 mL confirmed complete occlusion of SMA blood flow under fluoroscopic guidance, verifying the feasibility of intraoperative arterial blood flow control. SMA, superior mesenteric artery

Surgery was performed under general and epidural anesthesia with the patient in the supine position. Laparotomy through a full midline incision revealed a large tumor occupying the abdominal cavity. Elevation of the tumor provided adequate exposure of the feeding vessels (**Fig. 4A**).

**Fig. 4:**
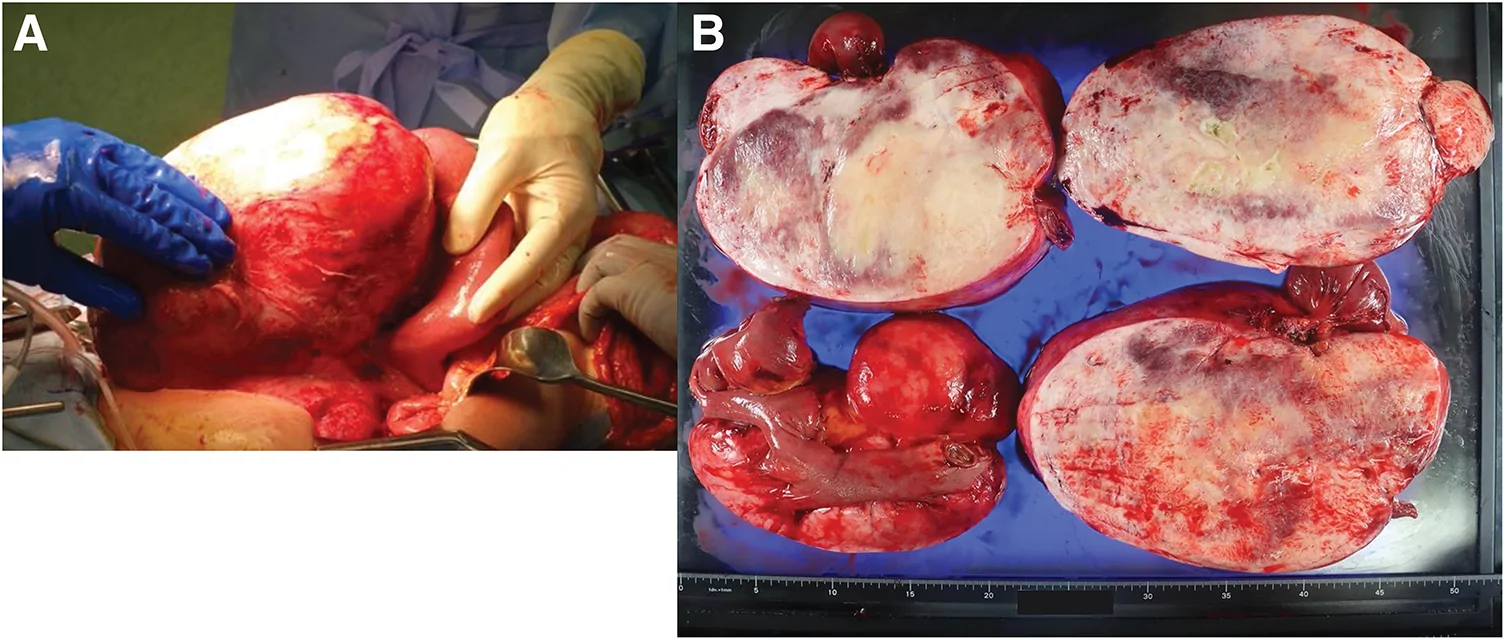
Intraoperative and macroscopic findings. (**A**) Following full midline laparotomy, the tumor is elevated to expose the extent of small bowel mesenteric involvement. (**B**) Cut surface of the resected specimen demonstrating a heterogeneous internal architecture.

The tumor involved the small bowel mesentery, transverse mesocolon, small intestine, and duodenum, requiring en bloc resection.

Before maneuvers carrying a risk of arterial bleeding, the anesthesiologist inflated the balloon at the surgeon’s direction, providing temporary control of proximal arterial inflow. The balloon was inflated once, for approximately 14 min. During and after occlusion, the surgeon confirmed that the color, peristalsis, and marginal arterial pulsation of the residual small intestine were well maintained. The tumor involved the SMA distal to the ICA bifurcation. Consequently, the SMA trunk together with the first and second jejunal arteries was ligated and divided.

As perfusion of the residual small bowel was expected to be maintained through the proximal jejunal branches and the marginal artery of the ICA, complete macroscopic resection was judged feasible. All lesions were resected *en bloc* along a dissection plane kept close to the tumor to preserve adjacent organs.

Following *en bloc* resection, approximately 200 cm of small bowel remained in continuity with the colon, and a side-to-side anastomosis was constructed between the descending duodenum and the jejunum. After reconstruction, indocyanine green fluorescence imaging confirmed adequate perfusion of the duodenal stump, residual small intestine, and colon. The balloon catheter was removed postoperatively by an interventional radiologist.

The operative time was 8 hours 43 minutes. During balloon occlusion, systolic blood pressure ranged from 85 to 109 mmHg and mean arterial pressure from 59 to 81 mmHg, and no continuous vasopressor support was required during occlusion or until tumor removal. Blood loss was 3380 mL, of which approximately 1850 mL occurred before balloon inflation and arose primarily from venous oozing from the extensive dissection surfaces. Intraoperative transfusion consisted of 6 units of packed red blood cells.

Arterial lactate remained within the normal range before and after occlusion.

The resected specimen measured 25 × 23 × 17 cm and weighed 5660 g (**Fig. 4B**). Histopathologic examination demonstrated fascicular proliferation of spindle-shaped cells with minimal pleomorphism in a collagenous background without mitotic figures or necrosis. Immunohistochemistry showed nuclear β-catenin positivity, confirming desmoid tumor. Negative surgical margins achieved R0 resection.

The postoperative course was free of major surgical complications, with no evidence of intestinal ischemia or catheter-related complications, such as thrombosis, dissection, or puncture-site complications. A transient paralytic ileus occurred and resolved with conservative management. The patient was discharged from the ICU on POD 1. No anastomotic leakage was detected on an oral contrast study performed on POD 8. Oral intake began on POD 14, and the patient was discharged on POD 24, with no signs of short bowel syndrome thereafter.

One year after surgery, MRI detected a 1.5-cm intra-abdominal recurrence in the right upper abdomen. Chemotherapy with MTX 30 mg and VBL 6 mg was initiated. Imaging demonstrated tumor shrinkage at 3 months, which was maintained through 7 months. However, treatment was discontinued because of Grade 2 nausea and vomiting according to CTCAE v5.0. As MRI demonstrated stable disease, close observation was selected. At 3 years postoperatively, no disease progression has been observed during follow-up.

## DISCUSSION

Desmoid tumors have an incidence of 2.4–4.3 cases per million population,^5)^ accounting for approximately 0.03% of all tumors.^1)^ Anatomically, they are classified as abdominal wall (49%), extra-abdominal (43%), and intra-abdominal (8%) tumors,^5)^ with intra-abdominal tumors being the least common and associated with a poorer prognosis.^4)^

Large intra-abdominal desmoid tumors are particularly rare. A PubMed search using the terms “(giant OR large) AND (intra-abdominal OR intraperitoneal) AND (desmoid tumor)” identified only 23 surgically resected cases, including the present case, with a maximum tumor diameter of ≥25 cm (**Table 1**).^6–27)^ Reported ages ranged from 14 to 68 years, with no clear predilection for either sex.

**Table 1:** Twenty-three cases of large intra-abdominal desmoid tumors (≥25 cm), including the present case

Author (year)^ref.^	Age	Sex	Risk factor	Max size (cm)	Adjacent or involved organs	Arterial hemorrhage control	Blood loss (mL)	F/U, months
Stürmlin (2012)^6)^	43	F	None	28	Omentum, spleen (caudal pole)	None	N/D	N/D
Tzvetanov (2012)^7)^	60	M	FAP, abdominal surgery	45	SMA/SMV trunk, mesenteric root, IVC, right kidney/ureter	None	2500	36
de Bree (2013)^8)^	31	F	Pregnancy	33	Transverse colon, transverse mesocolon, pancreas tail	None	N/D	54
Hanna (2016)^9)^	19	F	Pregnancy	30	Ileum	None	N/D	3
Williams (2016)^10)^	33	M	None	45	Omentum, ileocecum	None	N/D	N/D
Mizuta (2018)^11)^	17	M	None	30	Mesocolon, omentum, stomach, pancreas tail	N/D	220	N/D
Jin (2020)^12)^	28	F	FAP, pregnancy, abdominal surgery	30	None	None	N/D	6
Palacios-Fuenmayor (2020)^13)^	15	F	None	30	Right colon	None	N/D	21
Sioda (2020)^14)^	24	M	Abdominal trauma	31	Terminal ileum, cecum, ascending colon	None	N/D	3
Elhaddad (2022)^15)^	38	M	None	40	Ileocecum, appendix	None	N/D	5
Ichikawa (2022)^16)^	41	M	FAP	38	Abdominal wall, small intestine	Placement of a balloon catheter in the SMA	3430	22
Novotný (2022)^17)^	68	M	None	55	Small intestine, transverse colon	None	1000	5
Pujol-Cano (2022)^18)^	40	M	None	35	Stomach, transverse colon, omentum, pancreas, duodenum, mesentery, small bowel, spleen	None	N/D	2
Kim (2023)^19)^	18	M	None	38	Ileocecum	Embolization of a feeding vessel branching from SMA	3000	30
Rabai (2023)^20)^	14	F	None	30	Terminal ileum, cecum, ascending colon, appendix	N/D	N/D	12
Perdue (2023)^21)^	25	M	None	26	Right colon	None	N/D	N/D
Suzuki (2023)^22)^	20s	M	None	35	Transverse colon, duodenum, pancreas	None	570	12
Ghaddou (2024)^23)^	55	F	Pregnancy, abdominal surgery	25	Abdominal wall (rectus abdominis)	None	N/D	18
Medas (2024)^24)^	26	M	Abdominal surgery	40	Jejunum	None	N/D	48
Miyamoto (2024)^25)^	47	F	None	32	Ileum to mid-transverse colon, jejunum	None	N/D	48
Ibrahim (2025)^26)^	46	M	None	31	Ileum, cecum, mesentery, right ureter	None	N/D	9
Tanaka (2025)^27)^	21	M	None	38	Transverse colon, stomach, diaphragm	None	110	15
Present case	40	F	Pregnancy, abdominal surgery	25	Duodenum, small intestine	Placement of a balloon catheter in the SMA	3380	36

F, female; FAP, familial adenomatous polyposis; F/U, follow-up; IVC, inferior vena cava; M, male; N/D, no data; SMA, superior mesenteric artery; SMV, superior mesenteric vein

Among these cases, 3 were associated with FAP, 5 with pregnancy, 5 with prior abdominal surgery, and 1 with trauma, whereas most were sporadic without identifiable predisposing factors.

Although a direct causal relationship between cesarean section and desmoid tumor development remains unproven, mechanical stress from surgery has long been considered a contributing factor, suggesting that the previous cesarean sections may have been a plausible trigger in the present case.^2)^

Current guidelines recommend active surveillance as first-line management.^3)^ However, surgical resection should be considered for patients with abdominal symptoms, progressive tumor growth, or a risk of organ obstruction or perforation.^3)^

Resection of large intra-abdominal desmoid tumors is technically demanding because of their proximity to major vessels and invasion of adjacent organs. Involvement of the SMA trunk or its major branches substantially increases the risk of arterial bleeding.

Only 2 previous reports have described preoperative vascular interventions to control intraoperative arterial bleeding. Ichikawa et al. (2022) electively placed an SMA balloon catheter before surgery and inflated it when arterial bleeding occurred, resulting in blood loss of 3430 mL.^16)^ Kim et al. (2023) performed angiography-guided embolization of SMA feeding branches, with blood loss of 3000 mL.^19)^

Total blood loss in the present case was 3380 mL, comparable to these 2 reports (3000–3430 mL). Total blood loss is not a meaningful measure of this technique: in a tumor of this size, the extensive dissection surfaces inevitably produce a large volume of venous bleeding, which arterial occlusion cannot reduce. The value of the indwelling balloon lay in providing control of arterial inflow at the SMA origin, which could not be reached manually or with a clamp while the tumor occupied the abdominal cavity, thereby rendering potentially uncontrollable arterial bleeding immediately controllable during division of the encased jejunal arteries and SMA trunk. Balloon control by the anesthesiologist under the surgeon’s direction also enabled prompt hemorrhage management without requiring additional personnel.

## CONCLUSIONS

This report describes successful resection of a 25-cm intra-abdominal desmoid tumor using elective preoperative SMA balloon catheter placement. For large desmoid tumors with a high risk of arterial bleeding, preoperative vascular occlusion may facilitate intraoperative arterial-bleeding control and improve surgical safety.

## DECLARATIONS

### Use of artificial intelligence tools

DeepL (DeepL, Cologne, Germany) was used to translate reference articles into a Japanese draft for reading, and Claude (Anthropic, San Francisco, CA, USA) was used to assist in translating the Japanese draft of the manuscript into English. The authors reviewed and edited all outputs and take full responsibility for the content of the manuscript.

### Availability of data and materials

Further information regarding this case is available from the corresponding author upon reasonable request.
